# Assessing Starch Retrogradation from the Perspective of Particle Order

**DOI:** 10.3390/foods13060911

**Published:** 2024-03-17

**Authors:** Hao Lu, Jinling Zhan, Wangyang Shen, Rongrong Ma, Yaoqi Tian

**Affiliations:** 1 College of Food Science and Engineering, Northwest A&F University, Yangling 712100, China; 2 State Key Laboratory of Food Science and Resources, Jiangnan University, Wuxi 214122, China; 3 National Engineering Research Center of Cereal Fermentation and Food Biomanufacturing, Jiangnan University, Wuxi 214122, China; 4 School of Food Science and Engineering, Wuhan Polytechnic University, Wuhan 430023, China; 5 School of Food Science and Technology, Jiangnan University, 1800 Lihu Road, Wuxi 214122, China; 6 Analysis and Testing Center, Jiangnan University, Wuxi 214122, China

**Keywords:** small-angle X-ray scattering, starch, radius of gyration

## Abstract

Starch retrogradation is a complex process involving changes in the multi-scale structure. In particular, the particle order of retrograded starch is unclear. In this study, we measured the radius of gyration (R_g_) and radius of particles (R) of retrograded starch using small-angle X-ray scattering. Retrograded starch included various R_g_, and the values of R_g_ depended on the length and state of the starch chains. With time, the standard deviations of R decreased due to the increase in particle uniformity. Based on these results, a new method for assessing the degree of starch retrogradation was established from the perspective of the particle order. The accuracy of the new method was verified through differential scanning calorimetry and scanning electron microscopy. The microstructures of the samples indicated that the retrograded starch granules contained substructures (primary particles) of different sizes. This study provides a new perspective for analyzing the structure of retrograded starch.

## 1. Introduction

Starch, a massive biodegradable and renewable carbohydrate polymer, is the most common ingredient in food [1]. Starch retrogradation is the realignment of gelatinized starch chains (amylose and amylopectin), thereby affecting the characteristics of starch-based products [2]. The rearrangement of amylose chains occurs in the initial stage of retrogradation (short-term retrogradation), whereas the reassociation of amylopectin chains occurs after long-term storage [3]. Retrogradation is a complex process accompanied by a series of changes, such as gel formation and an increase in relative crystallinity, transforming multi-scale structures [4]. Unfolded amylose and amylopectin are arranged in an orderly manner during retrogradation [5]. The relative content of double helices and short-range order in wet starch noodles first increases and subsequently decreases [6]. Relative crystallinity reflects the change in the structure of retrograded starch from the perspective of crystals; the increased relative crystallinity of retrograded starch is due to the orientation of the crystalline structure and the perfection of crystallites [7,8]. The formation of crystals is related to the rearrangement of both amylose and amylopectin. During retrogradation, the nanoscale structure resulting from the self-assembly of amylose and amylopectin impacts the lamellar structure. There is a positive correlation between storage time and average lamellar thickness [9]. The crystallinity of starch-based foods plays a significant role in determining their properties. Crystallinity plays a crucial role in the in vitro digestibility of starch. In general, the digestibility of starch rises as its crystallinity increases [10]. Due to the low initial crystallinity of noodles, there is a slower rate of increase in the hardness of cooked noodles [11]. Processing can influence the crystallinity of starch. A decrease in starch crystallinity is commonly observed following heat treatment and physical damage [10,12]. The crystallinity of starch can be improved through heat moisture and annealing treatment [13]. The retrogradation of starch is affected by a variety of factors. In general, the retrogradation rate of amylose is higher than that of amylopectin [14]. Water plays a crucial role in the process of starch retrogradation. Due to the high moisture content, the starch molecular chains are further apart, making it difficult for starch retrogradation to occur. Additionally, the storage temperature has a notable effect on starch retrogradation. Starch nucleation is favored at 4 °C and the growth of starch crystal nuclei is ideal at a temperature of 25 °C [15]. During retrogradation, the entanglement of starch chains results in the formation of a particle structure [16]. However, there is currently a shortage of techniques for analyzing starch retrogradation from a particle viewpoint.

Small-angle X-ray scattering (SAXS) is a useful technique for studying multi-scale structures in materials [17]. The structure of carbon black was characterized using SAXS, and the multi-scale structure of carbon black, including continuum, cluster, network, particle, surface, and atoms, was represented in the scattering curve. Moreover, the “clusters” of carbon black were visualized as sphere-like objects with large radii filled with primary spherical particles of a small radius [18]. Starch chains intertwine and rearrange to form granule structures similar to clusters during retrogradation. Therefore, we hypothesized that the spherulite structures of retrograded starch were also filled with substructures (primary particles), that is, retrograded starch as micro-scale intact granules composed of nanoscale primary particles. The lengths of starch chains are different, and the size of the cluster substructure is affected by the length of these chains. In previous studies, the structure of retrograded starch has been elucidated using SAXS [9,19]. However, the changes in the order of the substructures (primary particles) of retrograded starch during retrogradation remain unclear. 

In this study, we studied the particle order and morphology of retrograded starch stored for different times using SAXS and scanning electron microscopy, respectively. Further, we established a new method for assessing the degree of retrogradation and verified its accuracy. 

## 2. Materials and Methods

### 2.1. Materials

Indica rice starch (IRS; amylose content: 17.9%) was obtained from Wuxi Jinnong Biotechnology Co., Ltd. (Wuxi, China). Amyloglucosidase (260 U/mL, A7095, EC 3.2.1.3), pancreatin (8 USP, P7545, EC 232-468-9), and isoamylase (500 U/mL, Cat. No. E-ISAMYHP) were purchased from Sigma-Aldrich Chemical Co., Ltd. (St. Louis, MO, USA). All other chemicals and reagents used were of analytical grade.

### 2.2. Preparation of Retrograded IRS

IRS was suspended (33%, *w*/*v*) in deionized water and heated in a boiling water bath for 30 min with stirring. When the gelatinized starch reached room temperature, 5 drops of 0.01‰ sodium azide were added as a preservative. The resulting starch paste was stored at 4 °C in several hermetically sealed 250 mL conical flasks. The starch samples stored for 0, 1, 3, 5, 7, 14, 21, 28, and 35 days are referred to as RIRS-0, -1, -3, -5, -7, -14, -21, -28, and -35, respectively. These starch samples were dried at 40 °C for 24 h. Dry starch was crushed and then passed through a 100-mesh sieve. The starch granules were then placed in a desiccator at 75% relative humidity for one week, and this relative humidity could be kept at 75% by utilizing saturated salt water. These samples were used for property characterization.

### 2.3. SAXS

Wet retrograde starch samples were prepared as described by Zhai et al. [20], with slight modifications. Starch slurries with a 75% water content were equilibrated overnight at 25 °C. SAXS measurements were performed using a Xeuss 3.0 C SAXS instrument (Xenocs, Grenoble, France). A wavelength of 1.54 Å was used with a sample–detector distance of 1070 nm. The scattering patterns were in the q range of 0.01–0.2 Å. Different R_g_ values were obtained using software (XSACTVersion 1.18).

### 2.4. High-Performance Anion-Exchange Chromatography (HPAEC)

The chain length distribution of the retrograded starch samples was measured using HPAEC with pulsed amperometric detection (HPAEC-PAD; ICS-5000+, Thermo Fisher Scientific, Massachusetts, USA), as described by Zhai et al. [20]. Briefly, 10 mg of retrograded starch samples was dispersed in a buffer with a pH of 4.5 at a concentration of 2 mg/mL. The mixtures were heated in boiling water for 30 min, and 4 μL of isoamylase was added to the gelatinized starch at 42 °C. The gelatinized starch samples were enzymatically hydrolyzed at 42 °C for 12 h. After the reaction, the enzyme was removed by heating and centrifugation. The supernatant was filtered through a 0.45 μm membrane with an injection volume of 5 μL at a flow rate of 0.4 mL/min using a linear gradient of NaOAc (0–400 mM) as the mobile phase.

### 2.5. Differential Scanning Calorimetry (DSC) 

The enthalpy change of the starch samples was determined using an X-DSC7000 calorimeter (TA Instruments, Delaware, USA). Briefly, 3–5 mg of dried sample powder and twice the mass of deionized water were added to an aluminum pan. The pans were sealed and equilibrated overnight. The pans containing the samples and an empty reference pan were loaded into the DSC instrument. Programmed scanning was performed as described in our previous study, and the retrogradation degree (RD) was calculated [21]. RD_ΔH_ was calculated according to the equation:

(1) $${\mathrm{RD}}_{\Delta \mathrm{Ht}}=\frac{{\Delta H}_{t}-{\Delta H}_{0}}{{\Delta H}_{\infty }-{\Delta H}_{0}}\;\times 100\%$$

where RD_ΔHt_ is the retrogradation degree (%) of the starch sample stored for t days, ΔH_0_ is the enthalpy change of the fresh gelatinized starch, ΔH_t_ is the enthalpy change of the starch sample stored for t days, and ΔH_∞_ is the enthalpy change of the retrograded starch stored for 35 days. 

### 2.6. Enzyme Hydrolysis of Retrograded Starch

To observe the morphologies of the retrograded starch samples, they were subjected to enzyme hydrolysis as per the method described by Chang et al. [22], with some modifications. Briefly, 200 mg of retrograded starch samples was added to a tube containing 4 mL of deionized water. Sodium acetate buffer (4 mL, pH 5.2) was added to the tubes, homogenized, and preheated to 37 °C with stirring at 160 rpm for 30 min. Pancreatin (2.25 g) was evenly dispersed in 20 mL of deionized water. The suspension was centrifuged at 5000 rpm for 10 min, and 13.5 mL of the supernatant was mixed with 1.6 mL of amyloglucosidase. Two milliliters of the mixed enzyme solution (pancreatin and amyloglucosidase) was then added to each tube. Then, the tubes were incubated in a water bath at 37 °C for 7 min, and aliquots of 0.1 mL were taken and mixed with 0.9 mL of 90% ethanol solution. After 7 min of incubation, the hydrolysate samples were centrifuged to remove the supernatant, and the sediments were washed twice with deionized water. The retentates were dried at 40 °C for 48 h, and the dried powder was used for morphological observations. 

### 2.7. Scanning Electron Microscopy (SEM)

The dried powder was glued to a conductive adhesive and coated with a thin gold film. The microstructures of the samples were observed at a resolution of 2000× using a scanning electron microscope (SU8100, JEOL, Tokyo, Japan).

### 2.8. Statistical Analysis

All data are reported as the mean ± standard deviation of triplicate experiments. All experiments were conducted in triplicate. The experimental data of a one-way analysis of variance (ANOVA) were analyzed using the SPSS 20.0 Statistical Software Program (SPSS Incorporated, Chicago, IL, USA).

## 3. Results and Discussion 

### 3.1. Nanoscale Lamellar Structure Analysis

**Figure 1:**
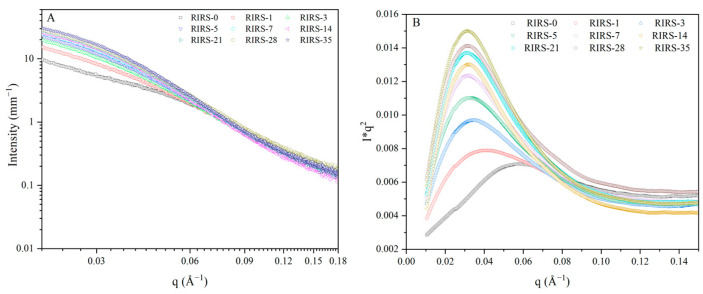
(**A**) SAXS curves of retrograded starch. (**B**) Lorentz-corrected SAXS patterns.

As shown in Figure 1A, no distinct peaks were observed for any of the retrograded starch samples, which is in line with the results of a previous study [9]. Lorentz-corrected SAXS patterns were obtained to observe the change in the scattering peak of the retrograded starch (Figure 1B). These peaks are related to nanoscale molecular organization, and the appearance of these peaks indicates the existence of periodic structures (amorphous and/or ordered structures) [23]. For retrograded starch, amylopectin and amylose can also be reassembled to form nanoscale periodic structures during storage [9,23]. The appearance of a retrograded starch peak is associated with this ordered aggregate structure. Furthermore, the peak position is related to the average lamellar repeat length in starch [24]. Compared to the peak position of the fresh gelatinized starch samples (RIRS-0), the peak positions of the other samples shifted to a lower-q range, indicating that the lamellar structure of the retrograded starch samples had changed. The peak position was negatively related to storage time, indicating that the thickness of the lamellar structure increased during storage. The size increased due to the aggregation and arrangement of amylopectin and amylose [9]. The peak position of the retrograded starch samples steeply shifted to the low-q range at the early stage of storage (0–7 days) and reached a plateau at the later stage (7–35 days). The first 7 days of retrogradation are characterized by the rapid aggregation and arrangement of amylose, after which, the retrogradation rate of amylopectin transitions to a slower rate [25]. In contrast, the number of unfolded and random starch chains was large at the initial stage, leading to drastic aggregation and an ordered arrangement. The structure of retrograded starch tends to stabilize at a later stage [26]. The retrogradation rate and enthalpy change of retrograded starch can provide similar information [27].

**Figure 2:**
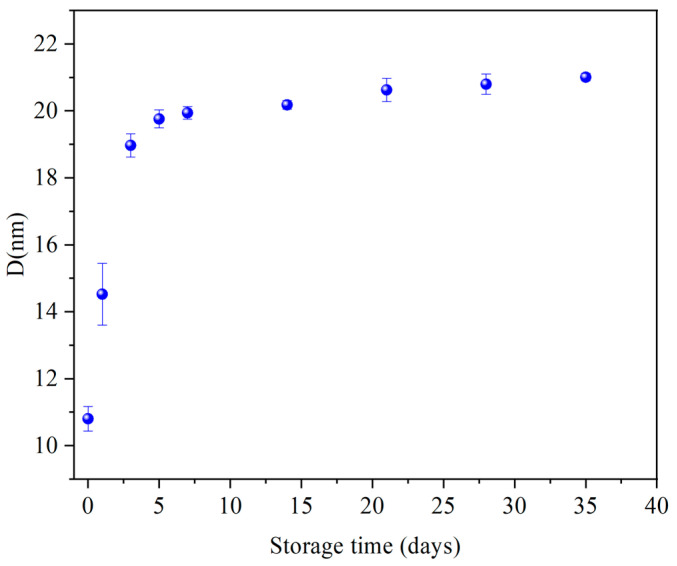
Lamellar thickness of retrograded starch.

The average lamellar thickness (D) of the starch samples was calculated using Bragg’s equation (D = 2π/q) [9,28,29]. A positive correlation was observed between the storage time and average lamellar thickness during retrogradation. The increase in the average lamellar thickness of the retrograded starch indicates that the size of the nanoscale structure changed because the order of the starch chains was enhanced by hydrogen bond interactions during retrogradation [30]. The starch chains were transformed from a disorganized radial arrangement to a vertically ordered arrangement. Therefore, the thickness of the lamellar structure increased. Similar results have been previously reported [9]. In particular, the rate of increase in the average lamellar repeat length during the initial storage period (0–7 days) was higher than that at the later stage (7–35 days). During the 0–7 days period, the average lamellar thickness grew from 10.80 to 19.94 nm, demonstrating an average daily increase of 1.31 nm. Over the course of 7–35 days, the average lamellar thickness experienced a mere 1.07 nm growth (Figure 2). This trend is attributed to the retrogradation of amylose and amylopectin. Stretched amylose can rapidly reassemble to form double helices as the seed nuclei for amylopectin. In contrast, the retrogradation rate of amylopectin is low [21]. 

### 3.2. Analysis of Particle Order

**Figure 3:**
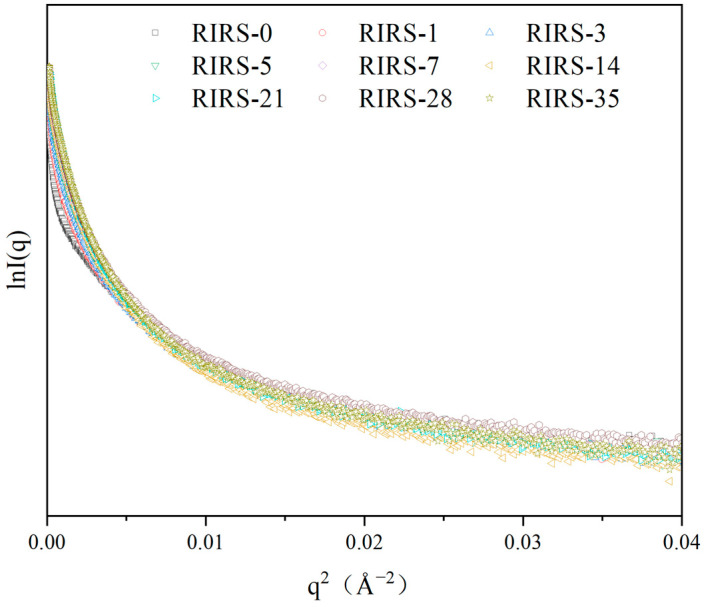
The Guinier curves of retrograded starch. RIRS-X: RIRS: retrograded indica rice starch; X: storage time.

R_g_ is the radius of gyration, which is the root-mean-square distance of the points of mass on the polymer chain from the center of gravity [31]. The compactness of the chain structure is evaluated using R_g_ [32] and can typically be obtained from the Guinier region. However, the curve in the Guinier region is not straight for a polydisperse system, and its slope is difficult to calculate (Figure 3). The experimental data can be interpreted using an approximate scattering function. In cases where the shifting process does not lead to a single straight line, the experimental curve can be broken into component curves, each of which can be shifted into a straight line [33]. Meanwhile, the scattering contributions of large and small particles are mainly in the low- and high-q ranges, respectively [28]. Combining the above theories, the Guinier curves of the retrograded starch samples were broken into component curves using the XSACT software (Version 1.18 ), and the R_g_ values were obtained. R_g_ was calculated according to previous studies with slight modifications [28,33,34,35,36]. The particle size distribution in the polydisperse system was obtained using an approximate scattering function, and the resulting data were an approximation. R_g_ was defined as R_g1_–R_g8_ based on the value of R_g_ and depended on the fitting range. The general agreement was that q×R_gmax_ should be less than 1.3, and high values were tolerated for globular objects [31,37] (XSACT software). The value of q×R_gmax_ (<2.5) was slightly higher than the reference value (1.3). Moreover, the value of χ^2^ was between 1.5 and 2.0, indicating an excellent fit to the experimental data [24]. The fitting ranges and R_g_ values were defined according to the above principles.

**Figure 4:**
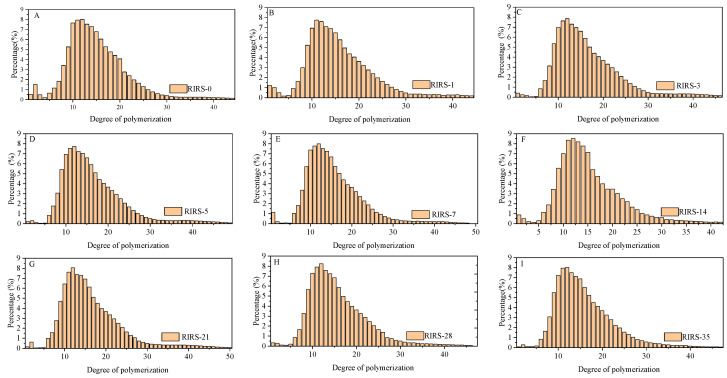
The chain length distribution of retrograded starch samples stored for different days (**A**–**I**).

Each retrograde starch sample had various R_g_ values due to the entanglement of starch chains of different lengths. The results of the chain length distribution demonstrated the existence of starch chains of various lengths (Figure 4). Long chains can form large particles. When the shape of the particles is the same, R_g_ is related to the size of the particles [28]. A large R_g_ is proposed to arise from a long starch chain and vice versa, and the self-entanglement of starch chains is ascribed to short- and long-range forces [26]. When starch chains transition from a single chain to an entangled state, the R_g_ value of the retrograded starch increases [26]. The R_g_ value decreases from the entanglement state to stabilization [26]. These results indicate that retrograded starch contains starch chains in various states. The variation trend of the R_g_ of different samples indicates that R_g7_ and R_g8_ can reflect the state of a single chain, and the entanglement state of starch chains is related to R_g3_–R_g6_. The values of R_g1_ and R_g2_ depend on the stabilization state of the starch chains. The percent reduction of R_g_ is only 3% from the entanglement state to stabilization, and the state of the chains changes during the process from disorder to order [26]. The formation of a stable structure of starch chains is attributed to crosslinking between the chains [26]. The increase in the value of the R_g_ of starch chains in a stable state was due to further crosslinking, and the values for R_g1_ and R_g2_ were higher than those for R_g3_–R_g6_.

**Figure 5:**
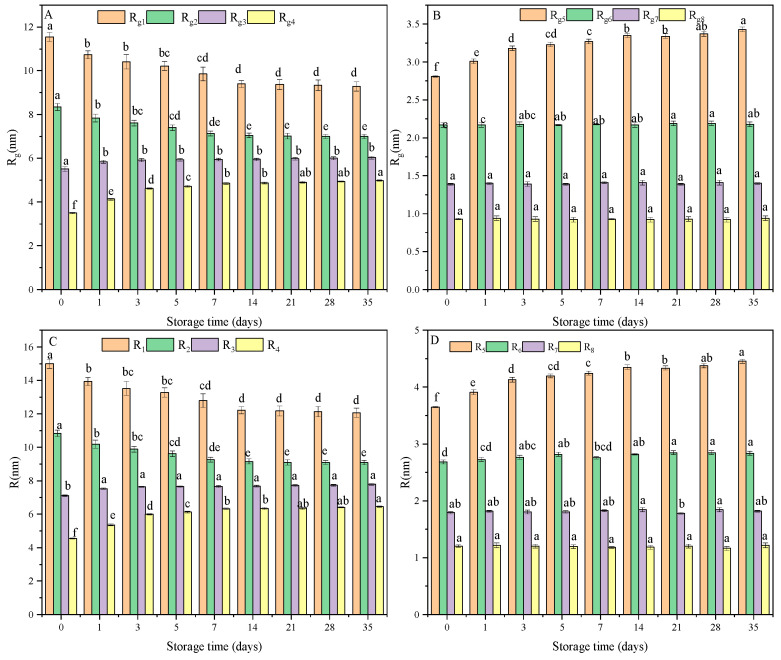
(**A**) The R_g1_–R_g4_ of retrograded starch samples. (**B**) The R_g5_–R_g8_ of retrograded starch samples. (**C**) The R_1_–R_4_ of retrograded starch samples. (**D**) The R_5_–R_8_ of retrograded starch samples. Values with different superscripts letters were significantly different.

As shown in Figure 5A,B, the same type R_g_ of the different samples displayed different variation trends during retrogradation. As the storage time increased (0–35 days), the reduction rates of R_g1_ and R_g2_ were approximately 19.50% and 16.18%, respectively, suggesting that the compactness of the retrograded starch structure increased [38]. The R_g3_–R_g6_ values of the different samples were positively related to storage time, because more starch chains were entangled. Furthermore, the values of R_g7_ and R_g8_ did not change significantly during retrogradation due to limited short- and long-range forces [26]. 

A previous study [18] showed that primary particles (substructures) of different sizes, composed of clusters of retrograded starch, are spherical. When the particle is spherical, the particle radius (R) can be calculated according to the equation (R_g_ = √0.6R), and the various R values of retrograded starch are presented in Figure 5C,D. With an extension of the storage time, the variation tendency of R was similar to that of R_g_, and the primary particles were referred to as P1–P8, according to the size of the particles. The R values of P7 and P8 remained unchanged with storage time, indicating that they might be the minimum particle reassembly units in retrograded starch. The reassembly of retrograded starch and the entanglement of starch chains increased the sizes of P3 and P6. In particular, the R_1_ and R_2_ of RIRS-0 were higher than those of RIRS-35, which seemed to be associated with increased compactness [26]. 

Although some values of D from the previous calculation were very close to the values of R, the two modes (particle mode and lamellae mode) were not contradictory. The two modes reflect the structure of the starch from different scales and perspectives. In other words, the primary particles aggregate and rearrange to form a lamellar structure. Similar structures have been proposed for native starch, where large and small blocklets are arranged in an orderly manner and stacked to form crystalline (hard shell) and semi-crystalline (soft shell) structures [39]. 

### 3.3. Evaluation of Retrogradation Degree (RD)

**Figure 6:**
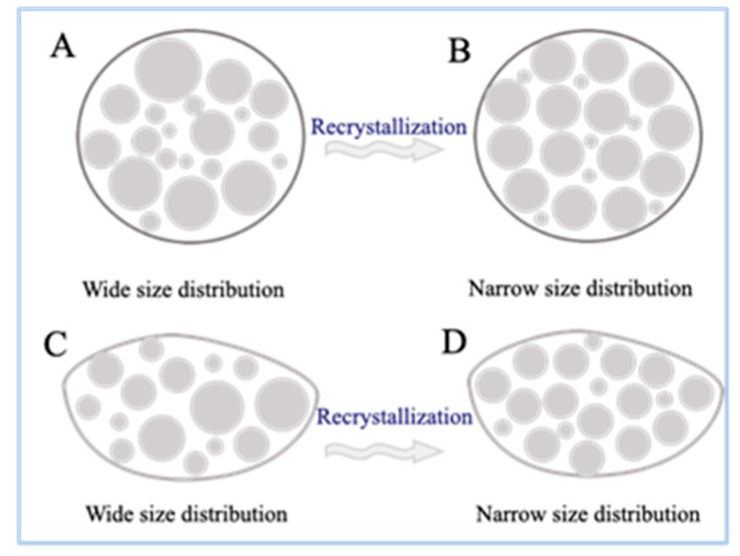
The cross-section views of particles. (**A**,**C**) Wide size distribution. (**B**,**D**) Narrow size distribution.

Based on the above results, a new method for assessing the RD of starch from the viewpoint of particle order was proposed. Several studies have reported that the order of retrograded starch increases during storage [40,41]. In these reports, the short- and long-range orders of retrograded starch were mainly assessed from the perspective of the helical structure. In this study, a new particle order was proposed. Uniform-sized particles are more likely to form ordered structures than wide particle size fractions, because small particles occupy the voids between large particles (Figure 6) [42]. The degree of dispersion of the data can be evaluated using standard deviation; the lower the standard deviation, the better the uniformity of the particles. The higher the particle order, the more energy required to destroy the structure, which is similar to the enthalpy change in the sample during DSC analysis. Based on the Avrami equation, the standard deviation of the particles can be used to evaluate the RD of the retrograded starch. 

**Figure 7:**
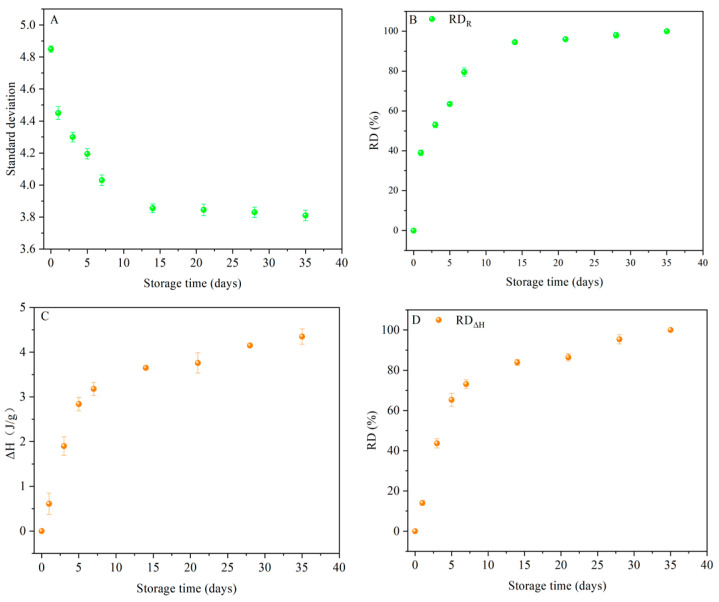
(**A**) The relationship between standard deviation of R and storage time; (**B**) the RD evaluated by standard deviation of R; (**C**) the relationship between ΔH and storage time; and (**D**) the RD evaluated by standard deviation of ΔH.

A scatter diagram of the standard deviation of R is shown in Figure 7A; as the storage time increased, the standard deviation of the particles decreased. According to the results, the particle order of the retrograded starch increased during starch retrogradation, indicating that the particle order was related to the degree of retrogradation. The longer the degree of retrogradation, the lower the standard deviation. Therefore, the standard deviation of R could be used to calculate the degree of retrogradation. Furthermore, when the particle is spherical, the particle radius (R) can be calculated according to the equation (R_g_ = √0.6R). The standard deviation of R_g_ reflects the degree of retrogradation. The RDR (RD obtained from R) was calculated according to the following equation [21]:

(2) $${\mathrm{RD}}_{\mathrm{Rt}}=\frac{{\Delta R}_{\mathrm{Rt}}-{\Delta R}_{R0}}{{\Delta R}_{R\infty }-{\Delta R}_{R0}}\times 100\%$$

where RD_Rt_ and ΔR_Rt_ are the retrogradation degree (%) and standard deviation of the starch samples stored for t days, respectively, and ΔR_R0_ and ΔR_∞_ are the standard deviations of RIRS-0 and RIRS-35, respectively. 

The first seven days of storage were characterized by a steeply increasing RD, after which, the retrogradation transitioned to a slower rate (Figure 7B). The movement of starch chains was violent during the initial stage (0–7 days), followed by a gentle change during the remaining time (7–35 days) [43]. Furthermore, the RD_R_ of RIRS-7 was as high as ~70%. 

To verify the accuracy of the standard deviation method for determining the RD of starch, the RD was characterized using the traditional DSC method. As expected, the RD_ΔH_ of the starch samples increased significantly during the first seven days of storage, followed by a more gradual increase in the remaining 28 days (Figure 7C,D). The values of the two types of RD (RD_R_ and RD_ΔH_) were different, because these values depend on the retrograded starch at different scale structures. ΔH describes the retrogradation of starch in terms of its helical structure. In contrast, the standard deviation for assessing starch retrogradation depends on the particle order. The parameters obtained from various scale structures were also different, as reported previously [9].

### 3.4. Recrystallization Kinetic Parameters

Moreover, the changes in the standard deviations of the R and ΔH of starch were fitted using the Avrami model, Equation (3) [44,45]:

X(t) = 1 − exp(−kt^n^) (3)

where X(t) is the RD on day t, k is the rate constant, and n is the Avrami exponent. The fitting results based on the Avrami equation are listed in Table 1. The fitting coefficients were greater than 0.97, indicating that these parameters obeyed the Avrami equation. The values of n obtained from the different methods were lower than 1, indicating that the nucleation mode was instantaneous [45]. These results illustrate the accuracy of the new method for assessing the RD of retrograded starch.

### 3.5. SEM 

**Figure 8:**
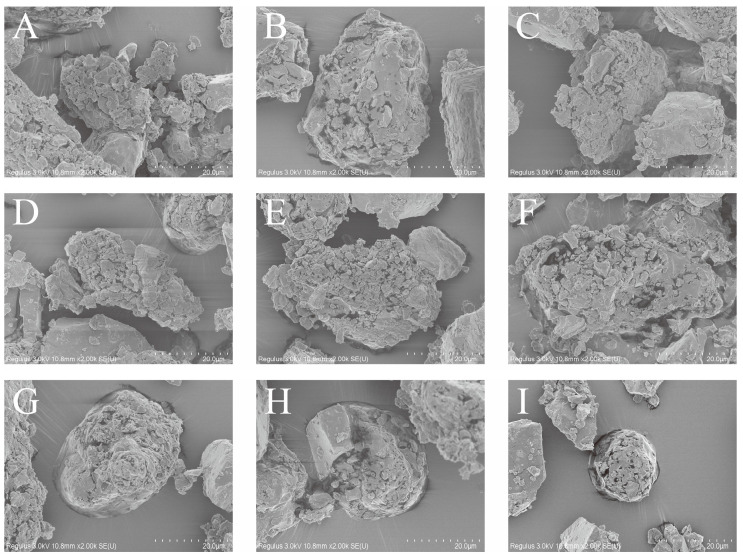
The SEM images of retrograded starch samples. (**A**–**I**) The images of RIRS-0, RIRS-1, RIRS-3, RIRS-5, RIRS-7, RIRS-14, RIRS-21, RIRS-28, and RIRS-35.

The morphologies of the samples subjected to enzymatic hydrolysis were observed to verify that the large granules were composed of small particles (Figure 8). If the enzymatic hydrolysis time was too long, the structure of starch was seriously damaged, which was not conducive to the observation of the granular structure. If the degree of damage was low, small particles were not easily observed, and the enzymatic hydrolysis of samples for 7 min could not only prove that the starch granules were composed of small particles, but could also be used for the measurement of the small particle size. Therefore, samples hydrolyzed for 7 min were used for the SEM analysis (Figure 8). Small particles of different sizes formed large starch granules, and micro- and nanoscale particles of spherical and irregular shapes were observed, supporting our hypotheses. In summary, the retrograded starch granules were composed of small spherical particles of different sizes.

## 4. Conclusions

In summary, a new method for evaluating starch retrogradation in terms of particle order was established. The values of R_g_ for the retrograded starch samples were different and related to the length and state of the starch chains. Large, micron-sized retrograded starch granules were composed of primary particles, as confirmed by SEM. Furthermore, the standard deviation of the different particles could reflect the RD of the retrograded starch. This method was found to be reliable by validating the DSC and Avrami equations. The SAXS results suggested that there was a particle order in the retrograded starch. In addition, there was a positive correlation between the particle order and retrogradation degree. According to the results mentioned above, SAXS has the potential to be utilized as a novel method in assessing the degree of starch retrogradation. Our new method provides a reference for regulating starch retrogradation from the perspective of particle order.

## Figures and Tables

**Table 1:** Recrystallization kinetic parameters obtained from different methods.

Method	k	n	R^2^
DSC	0.251 ± 0.012 ^b^	0.717 ± 0.014 ^b^	0.97
SAXS	0.357 ± 0.015 ^a^	0.980 ± 0.015 ^a^	0.99

Notes: DSC: Differential scanning calorimetry, SAXS: the radius of particle calculated from small-angle X-ray scattering. Values with different superscripts letters were significantly different.

## Data Availability

The original contributions presented in the study are included in the article, further inquiries can be directed to the corresponding author.
